# Negative Effect of Intravenous Antibiotics on Survival in Patients with Triple-Negative Breast Cancer

**DOI:** 10.3390/cancers17091498

**Published:** 2025-04-29

**Authors:** Stefan Lukac, Visnja Fink, Davut Dayan, Brigitte Rack, Wolfgang Janni, Krisztian Lato, Kristina Veselinovic, Sabine Heublein, Thomas Wolfram Paul Friedl, Elena Leinert

**Affiliations:** Department of Obstetrics and Gynecology, University Hospital Ulm, Prittwitzstrasse 43, 89075 Ulm, Germany

**Keywords:** triple-negative breast cancer, antibiotics, G-CSF, dose shift, dose reduction, febrile neutropenia, ECOG

## Abstract

Background: The anti-tumor response of the immune system is pivotal for treating triple-negative breast cancer (TNBC), particularly as targeted therapies are limited. However, the impact of immune-modulating factors such as the application of granulocyte-stimulating factors (G-CSFs) or infections, including febrile neutropenia, prophylactic or therapeutical application of oral antibiotics (OABs), and the need for intravenous antibiotics (IABs), on survival outcomes remains unclear. Methods: 1583 patients with early-stage TNBC enrolled in the SUCCESS A or C study underwent primary surgery, adjuvant chemotherapy, and radiotherapy if indicated. All patients had Eastern Cooperative Oncology Group (ECOG) status ≤ 2. The effects of G-CSF, OAB, and IAB application on overall survival (OS), invasive disease-free survival (iDFS), breast cancer-specific survival (BCSS), and distant disease-free survival (DDFS) were assessed. Results: Only IAB treatment was significantly associated with decreased survival in univariable analyses (OS: *p* = 0.003; iDFS: *p* = 0.036; BCSS: *p* = 0.011; DDFS: *p* = 0.044), while G-CSF and OAB administration were not. Adjusted multivariable Cox regressions including febrile neutropenia and dose reduction/shift, ECOG, age of patients, and other clinicopathological parameters confirmed a significant negative effect of IABs on OS (*p* = 0.020), BCSS (*p* = 0.018), and DDFS (*p* = 0.044). Conclusions: In summary, IABs during adjuvant chemotherapy seems to be a risk factor for inferior OS, BCSS, and DDFS in TNBC patients, possibly by affecting microbiome-related immune response modulation. Hence, preventive measures to avoid the need for IABs should be considered in these patients.

## Simple Summary

This study investigates the impact of immune-modulating factors, including granulocyte colony-stimulating factor (G-CSF) and antibiotic treatment during adjuvant chemotherapy, on survival outcomes in early-stage triple-negative breast cancer (TNBC) patients. A cohort of 1583 TNBC patients from the SUCCESS A and C trials, who underwent primary surgery, adjuvant chemotherapy, and radiotherapy, was analyzed. Univariable analysis identified intravenous antibiotic (IAB) treatment as significantly associated with worse overall survival (OS, *p* = 0.003), invasive disease-free survival (*p* = 0.036), breast cancer-specific survival (BCSS, *p* = 0.011), and distant disease-free survival (*p* = 0.044), whereas G-CSF and oral antibiotics showed no significant effects. Adjusted multivariable Cox regression confirmed IABs’ negative impact on OS (*p* = 0.020), BCSS (*p* = 0.018), and DDFS (*p* = 0.044). According to previous studies, one possible explanation might be microbiome-related immune modulation. Our findings highlight the need for preventive strategies to minimize IAB use during TNBC treatment, but further studies are required to evaluate underlying mechanisms.

## 1. Introduction

Breast cancer (BC) ranks as the most prevalent cancer among women [1], and it is typically categorized into four biological subtypes [2]. While specific treatment options such as endocrine therapy or targeted antibodies are available for luminal-like and human epidermal growth factor receptor-2 (HER2)-positive BC, chemotherapy has historically been the only systemic treatment option for triple-negative breast cancer (TNBC) [3]. Anthracyclines, cyclophosphamide, and fluorouracil, commonly used in TNBC treatment, exert immunostimulatory effects, inducing chemotherapy-induced cell death and overcoming immunological escape to reinvigorate attenuated functional immunity [4]. Recent advancements in TNBC therapy have seen the utilization of immune checkpoint inhibitors (ICIs) [5], although their efficacy varies in early-stage and metastatic TNBC [6,7], underscoring the importance of the antitumoral activity of the immune system, particularly cellular immunity [8]. Moreover, an increased number of tumor-infiltrating lymphocytes (TILs) is associated with a survival benefit in TNBC, contrasting with inferior survival in hormone receptor-positive breast cancer, further supporting the pivotal role of the immune system in combating TNBC [6,9,10]. However, one common side effect of systemic chemotherapy is myelotoxicity and febrile neutropenia, which can be mitigated by granulocyte-colony stimulating factors (G-CSFs) with or without oral antibiotics (OABs). In cases of febrile neutropenia or other severe infections during chemotherapy, hospitalization and intravenous antibiotics (IABs) are necessary. The question arises as to whether these interventions, which interact with the immune system, can affect outcomes in patients with TNBC.

## 2. Materials and Methods

The study population comprised all 1583 women with TNBC participating in one of the two large adjuvant breast cancer trials, SUCCESS A (EudraCT 2005-000490-21, NCT 02181101) and SUCCESS C (EudraCT 2008-005453-38, NCT 00847444), who received at least one dose of adjuvant chemotherapy. These studies recruited patients in 2005–2007 (SUCCESS A) and 2009–2011 (SUCCESS C) who had Eastern Cooperative Oncology Group (ECOG) status ≤ 2. Every patient underwent primary surgery with complete resection of the tumor followed by adjuvant chemotherapy and irradiation, if indicated, according to the respective study protocol. Possible chemotherapies were FEC-D (three cycles of 5-fluorouracil, epirubicin, and cyclophosphamide every 3 weeks, followed by three cycles of docetaxel every three weeks), FEC-DG (three cycles of 5-fluorouracil, epirubicin, and cyclophosphamide every 3 weeks, followed by three cycles of docetaxel plus gemcitabine every three weeks), or an anthracycline-free DC regime (six cycles of docetaxel and cyclophosphamide every three weeks). All patients provided written informed consent, and both SUCCESS A and SUCCESS C were conducted in accordance with Good Clinical Practice and the Declaration of Helsinki. A positive ethical approval from the respective leading ethics committee is available for both studies (SUCCESS A: Ludwig-Maximilian-Universität München 076/05; SUCCESS C: Heinrich-Heine-Universität Düsseldorf MC-LKP-319).

For each patient, age in years, weight in kilograms, and height in meters were documented and the body mass index (BMI) in kg/m^2^ was calculated. BMI was considered a categorical parameter and divided according to the classification of the World Health Organization into underweight, normal weight, overweight, and adiposity [11]. The histopathological parameters, including tumor stage (pT1, pT2, pT3, pT4), nodal status (pN0, pN1, pN2, pN3), histological grading (G1, G2, G3), hormone receptor status (positive, negative), HER2 status (positive, negative), and histologic type (ductal, lobular, other), were determined according to the standard histopathologic classification outlined in the study protocols. Additionally, patient and treatment factors such as ECOG score at the start of the first chemotherapy cycle, type of surgery (breast-conserving, mastectomy, other), combination of the chemotherapy as mentioned above, dose reduction (yes/no), dose shift of more than 6 days (yes/no), and incidence of febrile neutropenia during chemotherapy (yes/no), together with use of radiotherapy (yes/no), were recorded.

There was no primary recommendation for general G-CSF application in either study. The indication for G-CSFs were neutropenia (absolute neutrophile count < 0.5 × 10^9^/L) for more than 5 days, febrile neutropenia (neutropenia and temperature > 38.5 °C) requiring hospitalization and intravenous antibiotics), severe neutropenia (absolute neutrophile count < 0.1 × 10^9^/L), and necessity for dose shift due to insufficient leucocytes or neutrophils. OAB prophylaxis was indicated if the patient’s neutrophile granulocytes were <0.5 × 10^9^/L, with or without application of G-CSFs. According to the study protocol, the possible antibiotic drugs in this setting were 500 mg of levofloxacin once a day or 500 mg of ciprofloxacin twice a day. If the patient developed fever, especially around the expected nadir of the neutrophile granulocytes, hospitalization and application of IABs were indicated, and no oral antibiotic treatment was to be started in the case of febrile neutropenia.

The survival outcomes, including overall survival (OS), invasive disease-free survival (iDFS), breast cancer-specific survival (BCSS), and distant disease-free survival (DDFS), were determined using the standardized definitions for efficacy endpoints [12]. Overall survival (OS) considers all deaths as an event that occurred regardless of cause during the entire observation interval. For the calculation of invasive disease-free survival (iDFS), disease progression (local or metastatic) or patient death was counted as an event, whereas DCIS was not. For the analysis of breast cancer-specific survival (BCSS), only the death of a patient that was clearly attributable to breast cancer was considered an event. Distant disease-free survival (DDFS) includes the occurrence of distant metastasis or death as an event. Survival was calculated from the date of randomization until the earliest time of occurrence of an event or until the last comprehensive assessment for all survival analyses presented in this study. Patients without documented events were considered censored at the time of their last comprehensive assessment.

Patients’ characteristics reported in terms of categorical variables were reported in frequency tables with absolute numbers and percentages. Age (years) was the only continuous variable and reported as median and range. To reduce the number of items and avoid problems regarding statistical analyses caused by small subgroups, we combined categories of clinicopathological parameters for all subsequent analyses as follows: BMI (<25 vs. 25–29.99 vs. ≥30 kg/m^2^), tumor stage (pT1 vs. pT2 vs. pT3/pT4), nodal stage (pN0/pN1 vs. pN2/pN3), histological type (invasive ductal vs. invasive lobular/other), type of surgery (lumpectomy vs. mastectomy/other), and ECOG at start of first chemotherapy cycle (0 vs. ≥1). The associations between G-CSF, OAB, and IAB application and other clinicopathological parameters were analyzed by Chi-square tests for categorical parameters and Mann–Whitney U-tests for the continuous variable age. The strength of the pairwise associations among G-CSF, OAB, and IAB application (yes/no) was assessed using Cramer’s *V*, which ranges from 0 to 1 and measures how strongly two categorical nominal-scale variables are related. The survival endpoints OS, iDFS, BCSS, and DDFS were analyzed based on Kaplan–Meier estimates, illustrated using Kaplan–Meier curves, and compared for application of G-CSFs (yes vs. no), OABs (yes vs. no), and IABs (yes vs. no) using a log-rank test. Furthermore, univariable and multivariable Cox regression models adjusted by standard clinicopathologic parameters were used to analyze the effects of G-CSF, OAB, and IAB treatment on survival. In the multivariable Cox regression models, we included the known prognostic clinicopathological factors (age, menopausal status, BMI, pT, pN, grading, histological subtype), parameters related to interventions (irradiation therapy, type of surgery, type of chemotherapy, SUCCESS study), and variables that were significantly associated with G-CSF, OAB, or IAB application in the univariable analyses (ECOG at the start of the first chemotherapy cycle, dose reduction, dose shift of more than 6 days, febrile neutropenia). SPSS (Statistical Package for the Social Sciences) version 29 (SPSS Inc., Chicago, IL, USA) was used for data analysis. All *p*-values reported are two-sided, and α = 0.05 was applied as the significance level throughout; no adjustment was made to the significance level for multiple testing.

## 3. Results

The median age of the 1583 patients was 54 years (range 24–86), and the majority of the patients had pT1 or pT2 tumors (1525; 96.3%). Most patients (1081; 68.3%) had negative lymph nodes, and the dominating histological tumor type was ductal carcinoma (1369 patients, 86.5%). Loco-regional treatment consisted of lumpectomy in 1340 (84.6%) cases, and 1384 (87.4%) patients received irradiation therapy. At least one dose reduction was required in 220 (13.9%) patients, while for 309 (19.5%) patients at least one dose shift of more than 6 days was necessary. Febrile neutropenia occurred in 119 (7.5%) patients. More details on patient characteristics are presented in Table 1.

**Figure 1:**
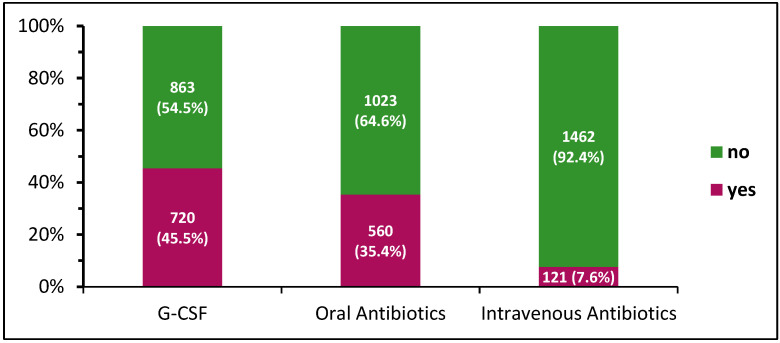
Interventions regarding immune system during adjuvant chemotherapy (*n* = 1583). G-CSFs: granulocyte-colony stimulating factors.

The most utilized intervention was the application of G-CSF, which was performed in 720 (45.5%) patients. OAB and IAB treatment was required in 560 (35.4%) and 121 (7.6%) patients, respectively (Figure 1). Overall, 650 (41.1%) patients received neither G-CSF nor oral or intravenous antibiotic intervention during adjuvant chemotherapy, 526 (33.2%) patients received only one of the three treatments, 346 (21.9%) patients received two different types of treatment, and 61 (3.9%) patients were treated with G-CSFs, OABs, and IABs (Table 2).

While G-CSF, OAB, and IAB application (yes/no) were significantly associated with each other (pairwise Chi-square tests, all *p* < 0.001), the strength of these associations among the application of the three treatment modalities of G-CSFs, OABs, and IABs was only low to moderate, as assessed by all Cramer’s *V* values for the pairwise comparisons being less than 0.3 (G-CSF and OAB: *V* = 0.287; G-CSF and IAB: *V* = 0.124; OAB and IAB: *V* = 0.210).

Patients that required G-CSF, OAB, or IAB application during adjuvant chemotherapy did not differ significantly from patients without G-CSF, OAB, or IAB application with regard to age (Mann–Whitney U-test; *p* = 0.242, *p* = 0.715, *p* = 0.263, respectively). G-CSFs were applied significantly more often in patients from the SUCCESS A study compared to patients from the SUCCESS C study. In addition, G-CSF application was associated with type of chemotherapy received, ECOG at the start of the first chemotherapy cycle, dose reductions, dose shifts of more than 6 days, and occurrence of febrile neutropenia (Table 3). Patients receiving oral or intravenous antibiotic treatment during chemotherapy had febrile neutropenia, dose reductions, and dose shifts of more than 6 days significantly more often than patients without antibiotic treatment. Overweight or adipose patients received intravenous antibiotic treatment significantly more often than normal-weight or underweight patients; in contrast, there were no significant associations between BMI and application of G-CSFs or oral antibiotics (Table 3). Patients with an ECOG score ≥1 at the start of the first chemotherapy cycle received intravenous antibiotic treatment significantly more often than patients with an ECOG score of 0.

**Figure 2:**
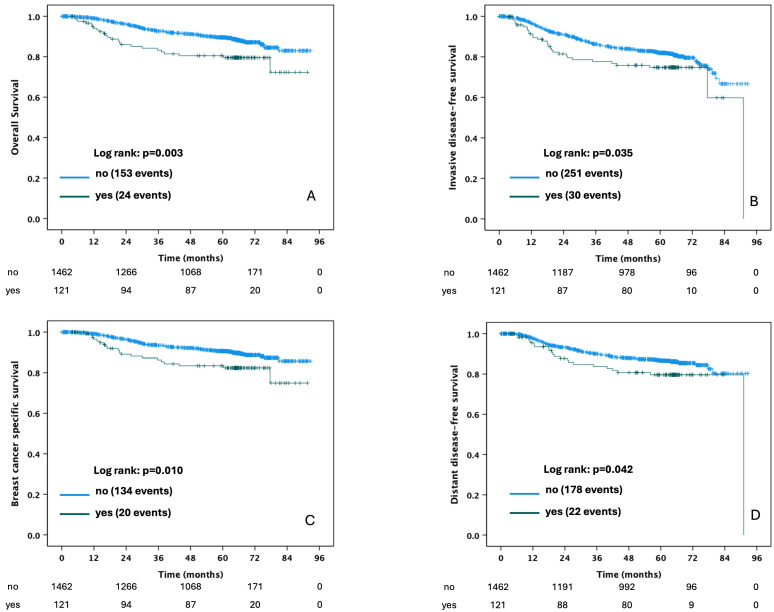
Kaplan–Meier curves according to the application of intravenous antibiotics during adjuvant chemotherapy (yes vs. no): overall survival (**A**), invasive disease-free survival (**B**), breast cancer-specific survival (**C**), and distant disease-free survival (**D**) in patients with triple-negative breast cancer. Patients with intravenous antibiotics *n* = 121; patients without intravenous antibiotics *n* = 1462.

For SUCCESS A, the median follow-up was 63.4 months (IQR 29.8), and for SUCCESS C, it was 64.3 months (IQR 19.2). Univariable Cox regressions showed no effect of G-CSF or OAB application on survival, while IAB application was significantly associated with worse outcome with regard to all four survival endpoints investigated. The respective hazard ratios are summarized in Table 4. Figure 2 shows the corresponding Kaplan–Meier curves for the four survival endpoints—OS, iDFS, BCSS, and DDFS—according to IAB application. Kaplan–Meier curves for the four survival endpoints according to G-CSF and OAB application are shown in Supplementary Figures S1 and S2, respectively.

A multivariable Cox regression analysis adjusted for all available clinicopathological parameters (see Table 5) confirmed a significant effect of IAB application on survival, with patients receiving intravenous antibiotic treatment during adjuvant chemotherapy having worse OS, BCSS, and DDFS compared to patients without the need for IAB treatment (OS: HR 1.81, 95% CI 1.10–2.98, *p* = 0.020; BCSS: HR 1.88, 95% CI 1.12–3.18, *p* = 0.018; DDFS HR 1.66, 95% CI 1.01–2.71, *p* = 0.044). G-CSF and oral antibiotic application were not significantly associated with any of the four survival endpoints in the adjusted multivariable analysis. The occurrence of febrile neutropenia, dose shifts of more than 6 days, or dose reductions during adjuvant chemotherapy were not significantly associated with survival outcome. Additional significant associations between other risk factors and one or more survival endpoints were found for age, BMI, tumor stage, nodal stage, and type of surgery (see Table 5).

## 4. Discussion

This study represents—to our knowledge—the first analysis of the impact of G-CSF, OAB, or IAB treatment on survival in a large cohort of patients with TNBC evaluated under the standardized conditions of a clinical trial.

As expected, the occurrence of febrile neutropenia during adjuvant chemotherapy was associated with the application of G-CSFs, OAB, and IABs in our study. In our cohort, only 7.5% of patients had febrile neutropenia, and 54.6% received G-CSFs, which is similar to previously published data [13,14]. American and European guidelines recommend primary prophylaxis of febrile neutropenia when its risk exceeds 20% based on patient factors and chemotherapy regimen [15,16]. It has been suggested that a machine learning approach can support the proper selection of patients by improving the prediction of febrile neutropenia [17]. Preventive application of G-CSFs and OABs has been confirmed as effective in avoiding febrile neutropenia during different chemotherapy regimens, but data regarding their impact on survival are lacking [18,19]. Our results indicate that febrile neutropenia does not worsen the survival of evaluated TNBC patients if properly treated.

The primary effect of G-CSFs is to increase neutrophil count. Furthermore, neutrophils play an essential role in fighting bacterial infections, which are more prevalent during chemotherapy, especially due to neutropenia [15,18]. According to the guidelines of the American Society of Clinical Oncology, there is no reported benefit in terms of iDFS or OS from applying G-CSFs in preventing dose reduction and maintaining dose intensity in any common cancer [16]. Our results are in line with and support these recommendations. Moreover, while the prognostic role of TILs in TNBC is well established [6,9,10], the role of neutrophils in combating TNBC remains puzzling [20]. Previous studies have shown that a higher minimum absolute lymphocyte count was associated with lower OS and BCSS in TNBC patients, but not neutrophil count [21]. It appears that, rather than absolute counts, the neutrophil-to-lymphocyte ratio may provide a more robust prediction of survival in TNBC patients [22]. This ratio is believed to reflect the balance between pro-tumoral inflammatory status and anti-tumoral immune response and may even predict response to therapy with ICIs in different tumors, including BC [23].

In our cohort, only IAB application was associated with worse outcome in terms of OS, BCSS, and DDFS, while OAB or G-CSF applications were not. Previous studies support the negative effect of antibiotics on survival in patients with TNBC, but none of the studies distinguished between IABs and OABs [24,25,26,27]. Ransohoff et al. retrospectively analyzed a database of 772 TNBC patients treated at two institutions regarding the effect of antimicrobial exposure on OS and BCSS [24]. They reported that cumulative antimicrobial exposure after the diagnosis of early-stage TNBC was associated with decreased OS and BCSS, consistent with our results [24]. ECOG, age, dose delays, and dose shifts were not significant in the multivariable analysis for OS or BCSS. Therefore, one possible explanation is that antimicrobial exposure before and during treatment is considered an immune-modulating risk factor for TNBC mortality [24]. The negative effect of concurrent exposure to antibiotics and ICIs was observed in 66 patients undergoing neoadjuvant therapy, including pembrolizumab, for HER2-negative early-stage BC [26]. Patients treated with antibiotics had a higher residual cancer burden and lower pathological complete remission rate [26]. Similarly, Xi Zhang retrospectively evaluated antibiotic administration during the first 30 days of neoadjuvant chemotherapy in 120 early-stage BC patients, with similar results: a lower rate of pathological complete remission and lower OS and iDFS in those treated with antibiotics [25]. Furthermore, antibiotic exposure not only during systemic treatment but also within six months before chemotherapy was associated with poorer survival in BC patients [28]. Worsened survival after antibiotic application has also been observed in other solid tumors, such as renal, lung, and esophageal cancer, but it does not seem to affect patients with hematological malignancies, such as acute myeloid leukemia [27,29].

In our study, the risk factors significantly associated with the need for IABs in univariable analyses were febrile neutropenia, dose reduction, dose shifts of more than 6 days, ECOG at the start of the first chemotherapy cycle, and BMI. Of these, only BMI also remained a significant factor negatively affecting survival (OS, iDFS, and BCSS) in the adjusted multivariable analysis. It is well known that increased BMI can worsen survival in BC due to its chronic inflammatory effect and differences in microbiome [30,31].

The gut microbiota plays a crucial role in human cancer [32,33], and bacterial colonization can affect the efficacy of chemotherapeutics [34]. Moreover, dysbiosis in the intestinal microbiome can compromise the efficacy of antitumor agents such as anthracyclines and 5-fluorouracil [35,36], which are used in BC treatment and influence the efficacy of ICI therapy against epithelial tumors [37]. Antibiotics have been shown to inhibit the clinical benefit of ICIs [37], but fecal microbiota transplant can overcome this resistance to ICIs in cancer patients [38]. Furthermore, the intestinal microbiota can influence responsiveness to anthracyclines, as shown in a rodent model of TNBC, and this effect can be modulated by antibiotics [35]. Taken together, the microbiome appears to be a crucial factor in the effectiveness of antitumoral treatment.

Thus, one possible explanation for the differing effects of IABs and OABs on survival observed in our study could be a different impact on the microbiome due to varying treatment durations, different classes of antibiotics used, and/or different pathways, depending on the route of administration. In the SUCCESS studies, OABs were used prophylactically in patients with severe neutropenia to prevent febrile neutropenia until the neutrophil count reached 0.5 × 10^9^ cells/L, indicating short use without involvement of a systemic inflammatory response. In contrast, according to the study protocol, the occurrence of fever prompted the use of IABs. The application of IABs is usually longer than that of prophylactic OAB and thus more likely to be accompanied by an inflammatory response due to the underlying infection. In addition to treatment duration, the category of the antibiotics used could also play a role. While fluoroquinolones were suggested for OAB, the specific IAB was not defined in the protocol. Typically, the IAB is administered during hospitalization and belongs to the category of broad-spectrum beta-lactam antibiotics, which are known to disturb the microbiome significantly more than fluoroquinolones [39]. Lastly, IABs are not subject to a first-pass effect in the liver, in contrast to OABs. Therefore, the different routes of administration may lead to differing concentrations of the antibiotics in the gut, potentially resulting in a more profound impact of IABs on the microbiome [40,41]. All these factors could contribute to our finding that only IABs significantly affected outcomes in our cohort.

Another aspect to consider is the interdependence of treatments with G-CSFs, OABs, and IABs. All three interventions are commonly administered in response to chemotherapy-induced neutropenia or its potential consequence: infection. G-CSFs are typically used prophylactically to mitigate neutropenia, while antibiotic use—particularly intravenous antibiotics—may reflect more severe infectious complications. This interdependence—even if accounted for in the adjusted multivariable Cox regressions—might complicate the interpretation of their individual effects on survival, as their administration is often driven by overlapping clinical indications and may be further affected by patient frailty and baseline vulnerability. However, as we included age and ECOG performance status at the start of the first chemotherapy cycle as factors in the multivariable analysis, patient frailty and baseline vulnerability were at least partly accounted for in our analysis.

Limitations of the study include the unknown duration and indications for antibiotic treatment, as well as the lack of recording the type of intravenous antibiotics used. In addition, at the time the SUCCESS A and C studies were conducted, ICIs were not yet approved as treatment option for TNBC, and patients only received standard anthracycline-containing or anthracycline-free chemotherapy treatments. However, the large cohort size, standardized treatment conditions, and inclusion of possible modifiers such as dose shift, dose reduction, ECOG, age, and febrile neutropenia contribute to the robustness of the results obtained in this study.

## 5. Conclusions

In conclusion, our study revealed worse OS, BCSS, and DDFS in early-stage TNBC patients treated with IABs during adjuvant chemotherapy, while neither the occurrence of febrile neutropenia nor OAB or G-CSF administration had an effect on survival in an adjusted multivariable analysis. Thus, prophylaxis of conditions such as febrile neutropenia and other infections requiring IABs in high-risk TNBC patients may be crucial and should be considered in order to avoid a possible deterioration of chemotherapy treatment effect caused by IAB application during the treatment phase.

## Figures and Tables

**Table 1:** Characteristics of clinicopathological parameters of the patients with triple-negative breast cancer included in the study.

		Count	Percentage (%)
**Total**		1583	100
**SUCCESS study**	SUCCESS A	735	46.4
	SUCCESS C	848	53.6
**Age [years]**	Median and range	54 (24–86)	
**Body mass index**	Underweight	21	1.3
	Normal weight	746	47.1
	Overweight	483	30.5
	Adiposity	333	21.0
**Menopausal state**	Premenopausal	647	40.9
	Postmenopausal	936	59.1
**Tumor stage**	pT1	805	50.9
	pT2	720	45.5
	pT3	45	2.8
	pT4	12	0.8
	Missing	1	0.1
**Nodal stage**	pN0	1081	68.3
	pN1	368	23.2
	pN2	94	5.9
	pN3	37	2.3
	Missing	3	0.2
**Histological type**	Invasive ductal	1369	86.5
	Invasive lobular	40	2.5
	Others	174	11.0
**Grading**	G1	13	0.8
	G2	272	17.2
	G3	1298	82.0
**Type of surgery**	Lumpectomy	1340	84.6
	Mastectomy	212	13.4
	Others	31	2.0
**Chemotherapy**	FEC-D	778	49.1
	FEC-DG	373	23.6
	DC	432	27.3
**ECOG at start of first chemotherapy cycle**	0	1239	78.3
	1	298	18.8
	≥2	4	0.3
	Missing	42	2.7
**Febrile neutropenia**	Yes	119	7.5
	No	1464	92.5
**Dose reduction**	Yes	220	13.9
	No	1363	86.1
**Dose shift (more than 6 days)**	Yes	309	19.5
	No	1274	80.5
**Irradiation therapy**	Yes	1384	87.4
	No	198	12.5
	Missing	1	0.1

**Table 2:** G-CSF application, OAB application, and IAB application received during adjuvant chemotherapy in patients with triple-negative breast cancer (*n* = 1583).

No Intervention	650 (41.4%)
G-CSFs only	337 (21.3%)
OABs only	173 (10.9%)
IABs only	16 (1.0%)
G-CSFs and OABs	302 (19.1%)
G-CSFs and IABs	20 (1.3%)
OABs and IABs	24 (1.5%)
G-CSFs, OABs, and IABs	61 (3.9%)

**Table 3:** Association of clinicopathological parameters of patients with triple-negative breast cancer with application of granulocyte-colony stimulating factors (G-CSFs), oral antibiotics (OABs), and intravenous antibiotics (IABs) during adjuvant chemotherapy.

	G-CSF Application			OAB Application			IAB Application		
	No (*n* = 863)	Yes (*n* = 720)	*p*-Value	No (*n* = 1023)	Yes (*n* = 560)	*p*-Value	No (*n* = 1462)	Yes (*n* = 121)	*p*-Value
**Age (median, range**)	53, 24–86	54, 25–79	0.242 ^§^	54, 26–78	53, 24–86	0.715 ^§^	53, 24–86	56, 30–77	0.263 ^§^
**SUCCESS Study**			0.007 ^#,^*			0.674 ^#^			0.546 ^#^
SUCCESS A	374 (43.3%)	361 (50.1%)		471 (46.0%)	264 (47.1%)		682 (46.6%)	53 (43.8%)	
SUCCESS C	489 (56.7%)	359 (49.9%)		552 (54.0%)	296 (52.9%)		780 (53.4%)	68 (56.2%)	
**Body mass index**			0.339 ^#^			0.799 ^#^			<0.001 ^#,^*
Underweight/normal weight	426 (49.4%)	341 (47.4%)		499 (48.8%)	268 (47.9%)		728 (49.8%)	39 (32.2%)	
Overweight	250 (29.0%)	233 (32.4%)		314 (30.7%)	169 (30.2%)		440 (30.1%)	43 (35.5%)	
Adiposity	187 (21.7%)	146 (20.3%)		210 (20.5%)	123 (22.0%)		294 (20.1%)	39 (32.2%)	
**Menopausal state**			0.247 ^#^			0.821 ^#^			0.506 ^#^
Premenopausal	364 (42.2%)	283 (39.3%)		416 (40.7%)	231 (41.2%)		601 (41.1%)	46 (38.0%)	
Postmenopausal	499 (57.8%)	437 (60.7%)		607 (59.3%)	329 (58.8%		861 (58.9%)	75 (62.0%)	
**Tumor stage**			0.998 ^#^			0.435 ^#^			0.084 ^#^
pT1	438 (50.8%)	367 (51.0%)		531 (51.9%)	274 (48.9%)		755 (51.6%)	50 (41.3%)	
pT2	393 (45.5%)	327 (45.4%)		457 (44.7%)	263 (47.0%)		655 (44.8%)	65 (53.7%)	
pT3/pT4	31 (3.6%)	26 (3.6%)		34 (3.3%)	23 (4.1%)		51 (3.5%)	6 (5.0%)	
Missing	1 (0.1%)	0 (0.0%)		1 (0.1%)	0 (0.0%)		1 (0.1%)	0 (0.0%)	
**Nodal stage**			0.304 ^#^			0.496 ^#^			0.991 ^#^
pN0/pN1	784 (90.8%)	665 (92.4%)		939 (91.8%)	510 (91.1%)		1338 (91.5%)	111 (91.7%)	
pN2/pN3	77 (8.9%)	54 (7.5%)		81 (7.9%)	50 (8.9%)		121 (8.3%)	10 (8.3%)	
Missing	2 (0.2%)	1 (0.1%)		3 (0.3%)	0 (0.0%)		3 (0.2%)	0 (0.0%)	
**Histological type**			0.069 ^#^			0.470 ^#^			0.228 ^#^
Invasive ductal	734 (85.1%)	635 (88.2%)		880 (86.0%)	489 (87.3%)		1260 (86.2%)	109 (90.1%)	
Invasive lobular/others	129 (14.9%)	85 (11.8%)		143 (14.0%)	71 (12.7%)		202 (13.8%)	12 (9.9%)	
**Grading**			0.634 ^#^			0.700 ^#^			0.765 ^#^
G1/G2	159 (18.4%)	126 (17.5%)		187 (18.3%)	98 (17.5%)		262 (17.9%)	23 (19.0%)	
G3	704 (81.6%)	594 (82.5%)		836 (81.7%)	462 (82.5%)		1200 (82.1%)	98 (81.0%)	
**Type of surgery**			0.295 ^#^			0.143 ^#^			0.348 ^#^
Lumpectomy	738 (85.5%)	602 (83.6%)		876 (85.6%)	464 (82.9%)		1234 (84.4%)	106 (87.6%)	
Mastectomy/others	125 (14.5%)	118 (16.4%)		147 (14.4%)	96 (17.1%)		228 (15.6%)	15 (12.4%)	
**Chemotherapy**			<0.001 ^#,^*			0.574 ^#^			0.784 ^#^
FEC-D	452 (52.4%)	326 (45.3%)		512 (50.0%)	266 (47.5%)		722 (49.4%)	56 (46.3%)	
FEC-DG	151 (17.5%)	222 (30.8%)		234 (22.9%)	139 (24.8%)		342 (23.4%)	31 (25.6%)	
DC	260 (30.1%)	172 (23.9%)		277 (27.1%)	155 (27.7%)		398 (27.2%)	34 (28.1%)	
**ECOG at start of first chemotherapy cycle**			<0.001 ^#,^*			0.126 ^#^			0.001 ^#,^*
0	703 (81.5%)	536 (74.4%)		813 (79.5%)	426 (76.1%)		1159 (79.3%)	80 (66.1%)	
≥1	134 (15.5%)	168 (23.3%)		184 (18.0%)	118 (21.1%)		266 (18.2%)	36 (29.8%)	
Missing	26 (3.0%)	16 (2.2%)		26 (2.5%)	16 (2.9%)		37 (2.5%)	5 (4.1%)	
**Febrile neutropenia**			<0.001 ^#,^*			<0.001 ^#,^*			<0.001 ^#,^*
Yes	31 (3.6%)	88 (12.2%)		38 (3.7%)	81 (14.5%)		86 (5.9%)	33 (27.3%)	
No	832 (96.4%)	632 (87.8%)		985 (96.3%)	479 (85.5%)		1376 (94.1%)	88 (72.7%)	
**Dose reduction**			<0.001 ^#,^*			<0.001 ^#,^*			<0.001 ^#,^*
Yes	96 (11.1%)	124 (17.2%)		115 (11.2%)	105 (18.8%)		185 (12.7%)	35 (28.9%)	
No	767 (88.9%)	596 (82.8%)		908 (88.8%)	455 (81.2%)		1277 (87.3%)	86 (71.1%)	
**Dose shift (more than 6 days)**			<0.001 ^#,^*			<0.001 ^#,^*			<0.001 ^#,^*
Yes	141 (16.3%)	168 (23.3%)		167 (16.3%)	142 (25.4%)		270 (18.5%)	39 (32.2%)	
No	722 (83.7%)	552 (76.7%)		856 (83.7%)	418 (74.6%)		1192 (81.5%)	82 (67.8%)	
**Irradiation therapy**			0.865 ^#^			0.333 ^#^			0.967 ^#^
Yes	753 (87.3%)	631 (87.6%)		888 (86.8%)	496 (88.6%)		1278 (87.4%)	106 (87.6%)	
No	109 (12.6%)	89 (12.4%)		134 (13.1%)	64 (11.4%)		183 (12.5%)	15 (12.4%)	
Missing	1 (0.1%)	0 (0.0%)		1 (0.1%)	0 (0.0%)		1 (0.1%)	0 (0.0%)	

* Significant results; ^#^ Chi square test without missing; ^§^ Mann–Whitney *U*-test.

**Table 4:** Univariable Cox regressions for the effect of G-CSF, OAB, and IAB application during adjuvant chemotherapy (yes vs. no) on survival parameters.

	Survival	HR 95%CI	*p*
**Granulocyte colony-stimulating factors**	Overall survival	0.98 (0.73–1.31)	0.879
(yes vs. no)	Invasive disease-free survival	0.93 (0.73–1.18)	0.538
	Breast cancer-specific survival	1.02 (0.74–1.40)	0.907
	Distant disease-free survival	0.91 (0.68–1.20)	0.481
**Oral antibiotics**	Overall survival	0,94 (0.69–1.28)	0.705
(yes vs. no)	Invasive disease-free survival	1.04 (0.82–1.32)	0.751
	Breast cancer-specific survival	1.00 (0.72–1.39)	0.996
	Distant disease-free survival	1.04 (0.78–1.39)	0.774
**Intravenous antibiotics**	Overall survival	1.92 (1.25–2.95)	0.003
(yes vs. no)	Invasive disease-free survival	1.50 (1.03–2.19)	0.036
	Breast cancer-specific survival	1.84 (1.15–2.95)	0.011
	Distant disease-free survival	1.58 (1.01–2.46)	0.044

**Table 5:** Multivariable adjusted Cox regression analysis of clinicopathological factors and their effect on survival parameters in patients with triple-negative breast cancer.

		OS		iDFS		BCSS		DDFS	
Parameter	Categories	Hazard Ratio (95% CI)	*p*	Hazard Ratio (95% CI)	*p*	Hazard Ratio (95% CI)	*p*	Hazard Ratio (95% CI)	*p*
**G-CSF**	Yes vs. no	0.98 (0.70–1.36)	0.891	0.91 (0.70–1.18)	0.469	1.03 (0.72–1.46)	0.886	0.85 (0.63–1.16)	0.311
**Oral antibiotics**	Yes vs. no	0.87 (0.62–1.23)	0.437	1.01 (0.77–1.33)	0.933	0.88 (0.61–1.26)	0.473	0.95 (0.69–1.31)	0.766
**Intravenous antibiotics**	Yes vs. no	1.81 (1.10–2.98)	0.020 *	1.37 (0.89–2.10)	0.153	1.88 (1.12–3.18)	0.018 *	1.66 (1.01–2.71)	0.044 *
**Febrile neutropenia**	Yes vs. no	0.92 (0.52–1.61)	0.762	0.96 (0.61–1.50)	0.848	0.92 (0.52–1.65)	0.788	0.86 (0.51–1.46)	0.584
**Age** (years)		1.00 (0.97–1.02)	0.654	0.99 (0.97–1.01)	0.187	0.98 (0.96–1.00)	0.108	0.98 (0.96–1.00)	0.037 *
**Menopausal status**	Postmenopausal vs. premenopausal	1.43 (0.87–2.38)	0.161	1.15 (0.78–1.71)	0.474	1.64 (0.96–2.77)	0.068	1.36 (0.86–2.15)	0.189
**Body mass index** (kg/m^2^)			0.003 *		0.027 *		0.011 *		0.060
	25–29.9 vs. <25	1.13 (0.77–1.64)	0.541	1.02 (0.76–1.37)	0.894	0.99 (0.66–1.48)	0.964	1.00 (0.71–1.41)	0.987
	≥30 vs. <25	1.87 (1.28–2.74)	0.001 *	1.47 (1.09–1.99)	0.013 *	1.71 (1.15–2.54)	0.008 *	1.48 (1.04–2.12)	0.031 *
**Tumor stage**			<0.001 *		<0.001 *		<0.001 *		0.018 *
	pT2 vs. pT1	1.87 (1.31–2.66)	<0.001 *	1.54 (1.18–2.01)	0.002 *	2.04 (1.40–2.98)	<0.001 *	1.56 (1.14–2.14)	0.006 *
	pT3/pT4 vs. pT1	2.67 (1.43–5.00)	0.002 *	2.57 (1.54–4.26)	<0.001 *	2.82 (1.43–5.56)	0.003 *	1.73 (0.91–3.26)	0.092
**Nodal stage**	pN2/pN3 vs. pN0/pN1	3.74 (2.57–5.46)	<0.001 *	3.24 (2.36–4.46)	<0.001 *	3.60 (2.39–5.41)	<0.001 *	3.57 (2.48–5.14)	<0.001 *
**Grading**	G3 vs. G1/G2	1.11 (0.75–1.64)	0.610	0.97 (0.72–1.32)	0.861	1.06 (0.70–1.60)	0.795	0.84 (0.59–1.19)	0.333
**Histological type**	Lobular/other vs. ductal	0.89 (0.56–1.42)	0.633	0.69 (0.46–1.03)	0.071	0.98 (0.60–1.58)	0.926	0.68 (0.42–1.11)	0.121
**Type of surgery**	Ablative/other vs. breast-conserving	1.43 (0.94–2.19)	0.096	1.27 (0.90–1.80)	0.176	1.45 (0.92–2.29)	0.109	1.79 (1.21–2.64)	0.003 *
**Chemotherapy**			0.648		0.899		0.421		0.627
	FEC-DOCG vs. FEC-DOC	0.92 (0.61–1.39)	0.683	0.94 (0.67–1.33)	0.735	0.91 (0.59–1.41)	0.678	0.89 (0.61–1.32)	0.573
	DOC-C vs. FEC-DOC	1.23 (0.77–1.97)	0.395	1.06 (0.75–1.50)	0.748	1.39 (0.83–2.32)	0.208	1.19 (0.77–1.84)	0.425
**Irradiation therapy**	Yes vs. no	0.69 (0.42–1.12)	0.135	0.74 (0.49–1.11)	0.148	0.88 (0.51–1.54)	0.659	1.27 (0.74–2.18)	0.390
**Success study**	SUCCESS C vs. SUCCESS A	0.66 (0.42–1.04)	0.073	0.96 (0.67–1.37)	0.823	0.61 (0.37–1.01)	0.053	0.73 (0.48–1.11)	0.143
**Dose reduction**	Yes vs. no	0.82 (0.51–1.31)	0.401	1.00 (0.70–1.42)	0.983	0.90 (0.55–1.47)	0.666	1.13 (0.75–1.71)	0.559
**Dose shift (more than 6 days)**	Yes vs. no	1.08 (0.74–1.57)	0.691	1.09 (0.81–1.47)	0.575	1.10 (0.74–1.64)	0.637	1.17 (0.82–1.66)	0.387
**ECOG at start of first chemotherapy cycle**	≥1 vs. 0	0.96 (0.66–1.40)	0.840	0.97 (0.72–1.30)	0.820	0.95 (0.64–1.42)	0.804	1.03 (0.73–1.46)	0.854

* Significant results; OS: overall survival, iDFS: invasive disease-free survival; BCSS: breast cancer-specific survival; DDFS: distant disease-free survival; G-CSFs: granulocyte-colony stimulating factors. F—fluorouracil, E—epirubicin, C—cyclophosphamide, D—docetaxel, G—gemcitabine; ECOG: Eastern Cooperative Oncology Group score.

## Data Availability

All data supporting the findings of this study are available within the paper.

## Supplementary Materials

The following supporting information can be downloaded at: https://www.mdpi.com/article/10.3390/cancers17091498/s1, Figure S1: Kaplan–Meier curves according to the application of GCSF during adjuvant chemotherapy (yes vs. no): overall survival (**A**), invasive disease-free survival (**B**), breast cancer-specific survival (**C**), and distant disease-free survival (**D**) in patients with triple-negative breast cancer. Figure S2: Kaplan–Meier curves according to the application of oral antibiotics during adjuvant chemotherapy (yes vs. no): overall survival (**A**), invasive disease-free survival (**B**), breast cancer-specific survival (**C**), and distant disease-free survival (**D**) in patients with triple-negative breast cancer.
