# Supplementary figures and images for: Negative Effect of Intravenous Antibiotics on Survival in Patients with Triple-Negative Breast Cancer
Source: Cancers (Basel). 2025 Apr 29;17(9):1498. doi: 10.3390/cancers17091498 (PMC12071010; doi:10.3390/cancers17091498)

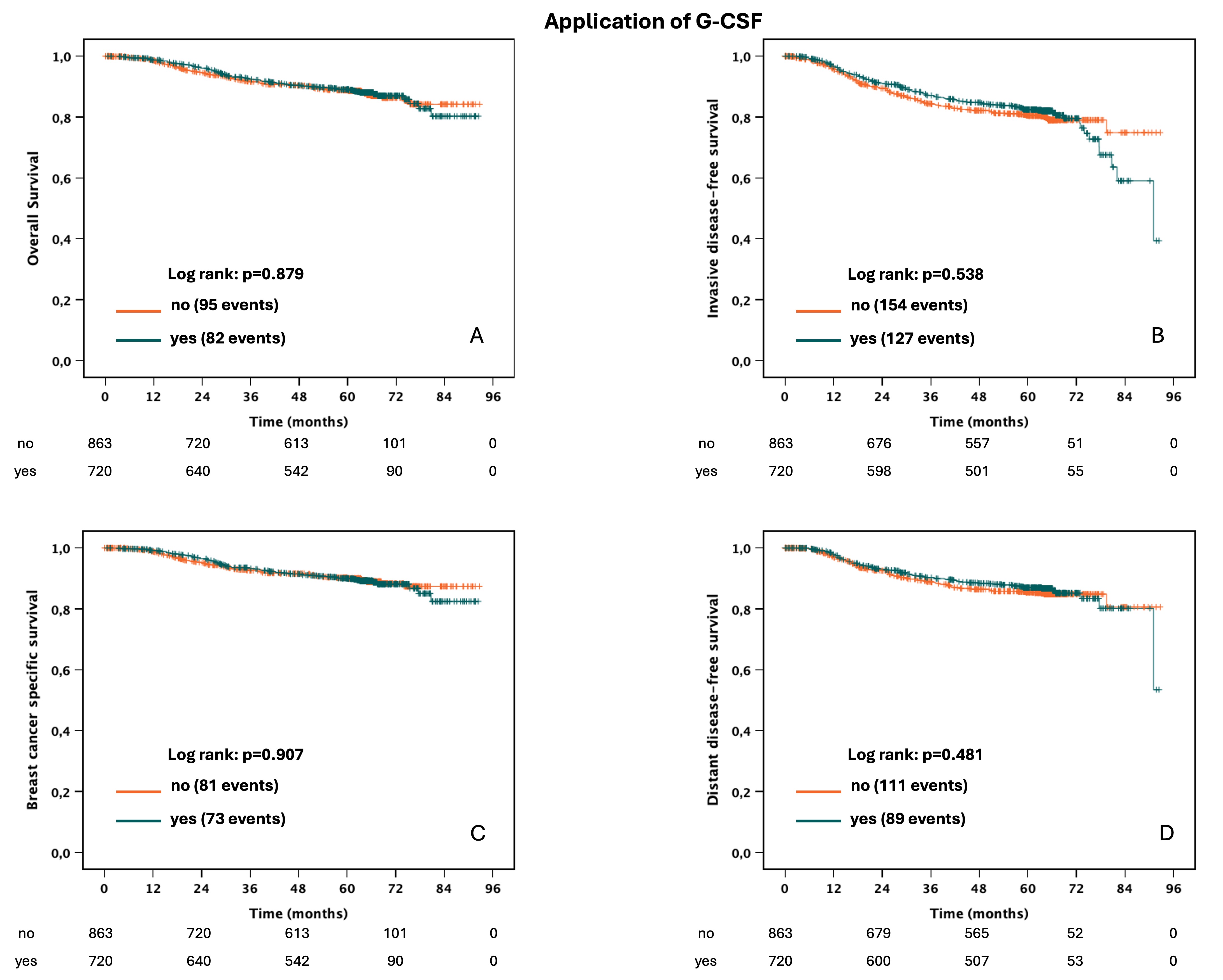

Supplement: Supplementary file 1 [file cancers-17-01498-s001.zip › Figure S1-GCSF_Survival.jpg]

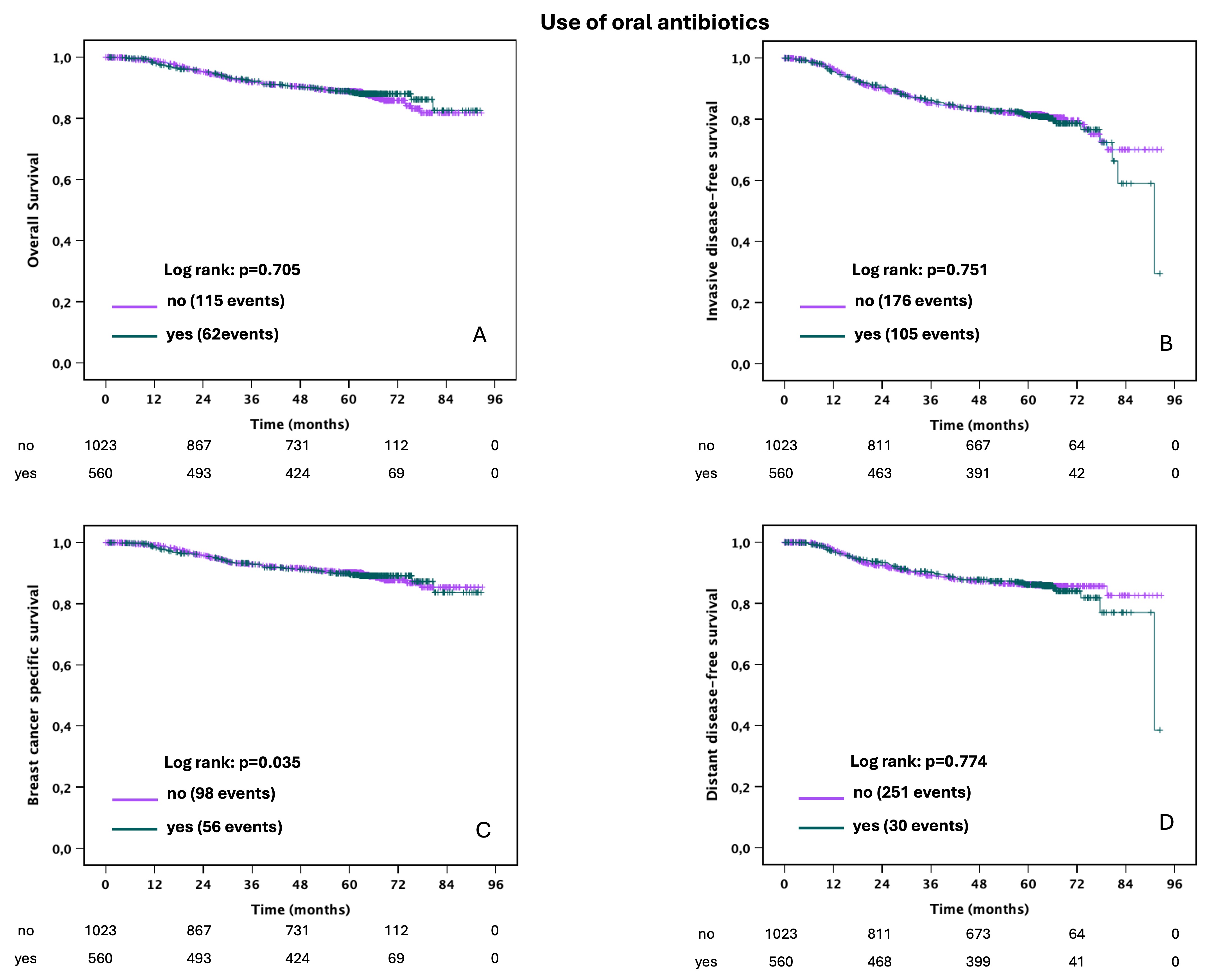

Supplement: Supplementary file 1 [file cancers-17-01498-s001.zip › Figure S2-OAB_Survival.jpg]
